# 18F-NaF Positive Bone Metastases of Non 18F-FDG Avid Mucinous Gastric Cancer

**DOI:** 10.4274/mirt.92400

**Published:** 2015-11-02

**Authors:** Çiğdem Soydal, Elgin Özkan, Özlem Nuriye Küçük, Metin Kemal Kır

**Affiliations:** 1 Ankara University Faculty of Medicine, Department of Nuclear Medicine, Ankara, Turkey

**Keywords:** Gastric cancer, bone metastasis, positron emission tomography/computed tomography

## Abstract

Detection of gastric cancer bone metastasis is crucial since its presence is an independent prognostic factor. In this case report, we would like to present 18F-NaF positive bone metastases of non 18F-FDG avid gastric mucinous cancer.

## Abstract (tr)

Mide kanserinin kemik metastazının saptanması bağımsız bir prognostik faktör olması nedeni ile kritiktir. Bu olguda 18F-FDG tutulumu izlenmeyen müsinöz mide kanserine ait 18F-NaF pozitif kemik metastazlarını sunmak istedik.

## INTRODUCTION

Gastric cancer could metastasize to different sites prior to diagnosis. The rate of bone metastasis has been reported as 1% to 20% for gastric cancer (1,2,3). Detection of gastric cancer bone metastasis is crucial since its presence is an independent prognostic factor (4). In this report, we would like to present a case with gastric cancer bone metastases that could not be shown by 18F-FDG positron emission tomography/computed tomography (PET/CT).

## CASE REPORT

**Figure 1:**
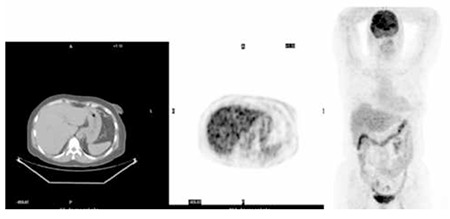
Axial computed tomography, PET and whole body maximum intensity projection 18F-FDG positron emission tomography/computed tomography images of the patient. There was no pathological uptake in the stomach or entire body

**Figure 2:**
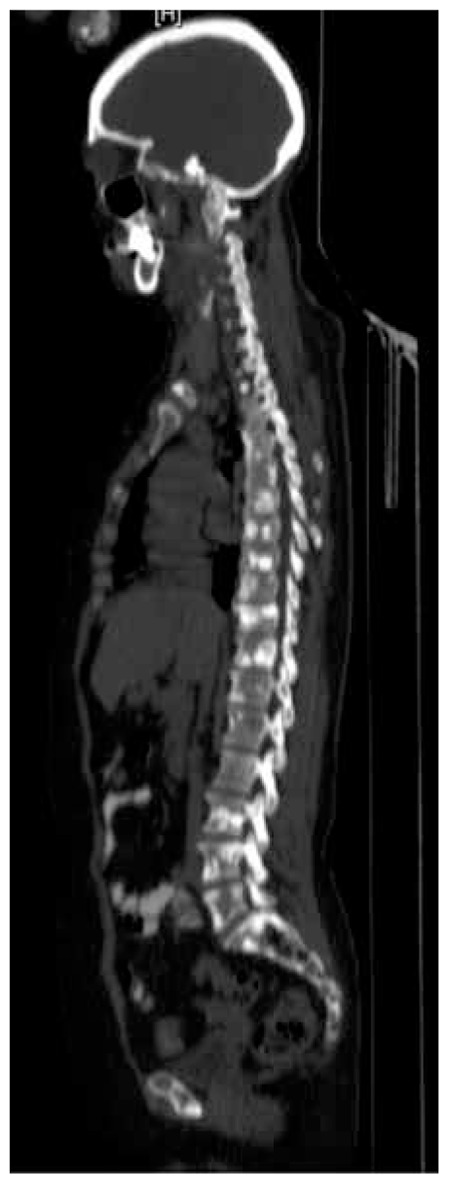
Sagittal computed tomography image of the patient. Multiple sclerotic lesions were seen in the entire skeleton

**Figure 3:**
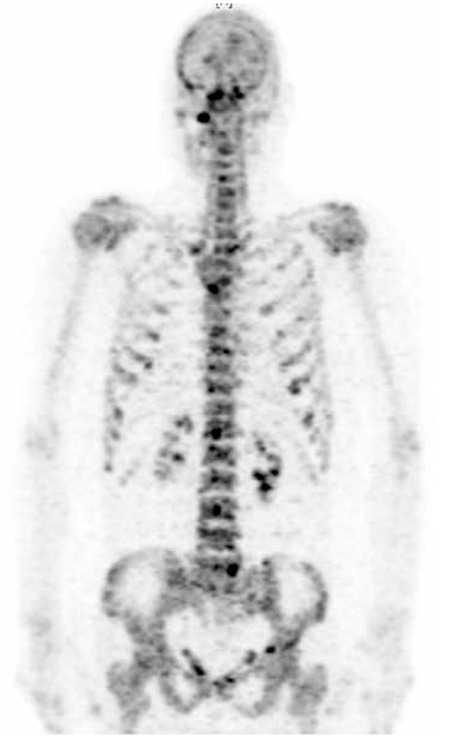
Whole body maximum intensity projection 18F-NaF positron emission tomography/computed tomography image of the patient. Intense 18F-NaF uptake was seen in sclerotic bone lesions

A 58 years old female patient with histopahologically proven gastric mucinous adenocarcinoma was referred to Ankara University Medical Faculty Department of Nuclear Medicine with a request of 18F-FDG PET/CT for staging. The whole body 18F-FDG PET/CT imaging was performed approximately 1 hour after intravenous injection of 370 MBq 18F-FDG. PET/CT images were acquired with GE Discovery ST PET/CT scanner (General Electric, Milwaukee, Wisconsin, USA). Emission PET images were reconstructed with non-contrast CT data for attenuation correction. In the evaluation of 18F-FDG PET/CT images, there was no pathological uptake in the stomach and whole body except diffuse increase in gastric wall thickness (Figure 1). Multiple sclerotic bone lesions were detected in axial CT images (Figure 2). An 18F-NaF PET/CT was performed to exclude bone metastases. 18F-NaF PET/CT images were obtained by the same scanner and parameters with CT, approximately 30 minutes after intravenous injection of 135 MBq 18F-NaF from vertex to feet. Intense 18F-NaF uptake was seen in multiple sclerotic bone lesions in the vertebral column, sternum, ribs, scalp and both scapula (Figure 3).

## LITERATURE REVIEW AND DISCUSSION

18F-FDG PET/CT is a hybrid imaging modality used in the staging of several cancers. However, the role of 18F-FDG PET/CT in the detection of bone metastases of gastric cancer is controversial (4,5,6,7,8,9,10). No algorithm has yet been defined to detect bone metastases of gastric cancer.

In our case, bone metastases of gastric cancer could not be shown by 18F-FDG PET/CT. In our case, we suspected bone metastases of gastric cancer in spite of lack of 18F-FDG uptake, because absence of uptake in the primary tumor was probably related to the mucinous component and sclerotic pattern of bone lesions. For these reasons an 18F-NaF PET/CT was performed to evaluate bone lesions, and 18F-NaF PET/CT confirmed bone metastases.

Conventional staging modalities such as bone scintigraphy are more valuable especially in patients with non 18F-FDG avid tumors. Various imaging methods including 18F-FDG PET/CT, whole body bone scintigraphy, magnetic resonance imaging and CT could be utilized to detect bone metastases. Tc-99m MDP bone scintigraphy is the traditional method to evaluate bone metastases of several cancers with low cost (5). The poor spatial resolution and longer duration of the examination result in limitations to bone scintigraphy. High quality images of the skeleton can be obtained within one hour after intravenous injection of 18F-NaF (6). In a recent study, Iagaru et al. (7) have reported that 18F-NaF PET/CT is superior to 18F-FDG PET/CT in the detection of bone metastases. An advantage of combined PET/CT systems is that they provide skeletal system evaluation with highly sensitive and specific images (8). During evaluation of 18F-FDG PET/CT images, skeletal lesions could be seen in CT series and these lesions could be evaluated by other methods to show bone metastases. In patients with non 18F-FDG avid tumors that could often metastasize to bone, CT series should be carefully evaluated to search bone lesions.

**Informed Consent:** It was taken, **Concept:** Çiğdem Soydal, **Design:** Çiğdem Soydal, **Data Collection or Processing:** Elgin Özkan, **Analysis or Interpretation:** Çiğdem Soydal, Özlem Nuriye Küçük, **Literature Search:** Çiğdem Soydal, Metin Kemal Kır, **Writing:** Çiğdem Soydal, **Peer-review:** Externally peer-reviewed, **Conflict of Interest:** No conflict of interest was declared by the authors, **Financial Disclosure:** The authors declared that this study has received no financial support.
